# Superoxide Flashes in Mouse Skeletal Muscle Are Produced by Discrete Arrays of Active Mitochondria Operating Coherently

**DOI:** 10.1371/journal.pone.0013035

**Published:** 2010-09-28

**Authors:** Sandrine Pouvreau

**Affiliations:** Physiologie Intégrative, Cellulaire et Moléculaire, Université Lyon 1, UMR CNRS 5123, Villeurbanne, France; Hospital 12 Octubre Madrid, Spain

## Abstract

Reactive Oxygen Species (ROS) constitute important intracellular signaling molecules. Mitochondria are admitted sources of ROS, especially of superoxide anions through the electron transport chain. Here the mitochondria-targeted ratiometric pericam (RPmt) was used as a superoxide biosensor, by appropriate choice of the excitation wavelength. RPmt was transfected *in vivo* into mouse muscles. Confocal imaging of isolated muscle fibers reveals spontaneous flashes of RPmt fluorescence. Flashes correspond to increases in superoxide production, as shown by simultaneous recordings of the fluorescence from MitoSox, a mitochondrial superoxide probe. Flashes occur in all subcellular populations of mitochondria. Spatial analysis of the flashes pattern over time revealed that arrays of mitochondria work as well-defined superoxide-production-units. Increase of superoxide production at the muscle fiber level involves recruitment of supplemental units with no increase in per-unit production. Altogether, these results demonstrate that superoxide flashes in muscle fibers correspond to physiological signals linked to mitochondrial metabolism. They also suggest that superoxide, or one of its derivatives, modulates its own production at the mitochondrial level.

## Introduction

Reactive oxygen species (ROS) refer to a group of oxygen containing molecules having the capability of reacting with reduced compounds. They were once viewed only as harmful molecules involved in disease and aging. Nowadays, this early perception is being replaced by a new concept whereby at low concentration ROS serve a physiological role as signaling molecules, while at high concentration they can damage critical cellular components, inducing cell death.

This dual role of ROS is fundamental in skeletal muscle physiology/pathology. On the one hand, ROS have been postulated to be involved in a panel of distinct muscle disorders, such as myotonic dystrophy, Duchenne dystrophy, central core disease, malignant hyperthermia and muscle fatigue [1], [2]. On the other hand, numerous evidences support a role of ROS in several muscle functions, such as force production [3], initiation of adaptative changes in gene expression [4], and regulation of calcium channels, calcium transporters and calcium-sensing proteins [5]–[7].

Several sources of ROS are present in mammalian skeletal muscle [4], [6], [8] the main produced species being superoxide anions (O_2_^−^) and hydrogen peroxide (H_2_O_2_). Mitochondria are often cited as the major site of superoxide production in tissues. Superoxide is generated from molecular O_2_ by the mitochondrial electron transport chain (ETC) and several other enzymes. ROS production by mitochondria is most likely of primary importance in skeletal muscle as these organelles are in close apposition with calcium stores [9] containing redox –sensitive calcium-signaling proteins. Indeed, close location between ROS source and target is mandatory since efficient cellular antioxidant defenses and ROS own reactivity restrain their spatial diffusion.

The recent identification of brief bursts of superoxide production -superoxide flashes- within the matrix of mitochondria in resting cells opened up a completely new area into ROS signaling studies [10]. Superoxide flashes have been described in a wide range of cells, including cardiac myocytes, skeletal myotubes, neurons, neuroendocrine cells and clonal cell lines.

In the present work, the mitochondria-targeted calcium biosensor ratiometric-pericam-mt (RPmt) [11] was transfected into mouse skeletal muscle fibers for use as a superoxide biosensor, by appropriate choice of the excitation wavelength. Results show that 1) Superoxide flashes in adult skeletal muscle fibers are physiological events linked to metabolism and involving the ETC. 2) Mitochondria operate as well defined units for the production of superoxide, active units being adjacent to quiescent ones. 3) Superoxide flashes cause depolarizations of mitochondria and releases of calcium. These phenomenons involve channels distinct from the classical permeability transition pore (PtP) and inner membrane anion channel (IMAC). 4) Increase of superoxide production at the fiber level is mediated by a recruitment of mitochondrial units and not by an increase of the production per unit. Superoxide or one of its derivatives may be involved in the activation of quiescent mitochondria.

## Results

### Targeting of RPmt

**Figure 1:**
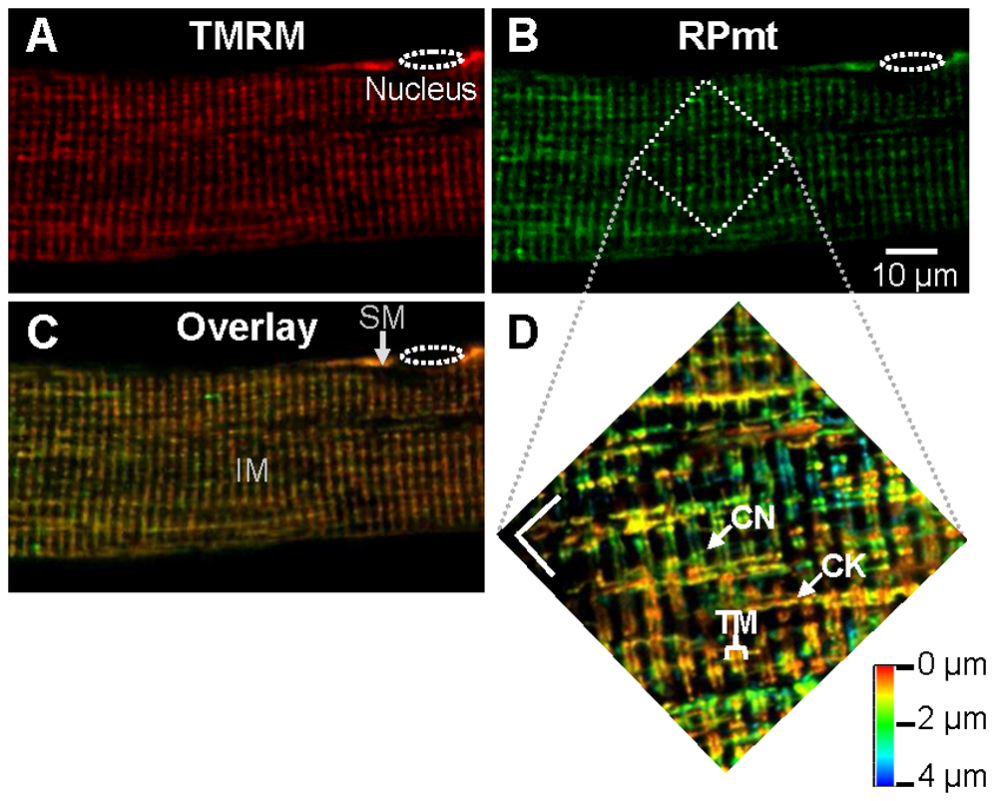
Targeting of RPmt to mitochondria. **A**, **B** Confocal images (x,y) of fluorescence of a skeletal muscle fiber transfected with RPmt (B) and loaded with TMRM (A). Images are averaged 4 times in line mode. **C** Overlay, showing the colocalisation of the two fluorescence patterns. SM: subsarcolemmal mitochondria; IM: intermyofibrillar mitochondria. **D** 3D reconstruction of a 4 µm-thick slice of the mitochondrial network. Images are averaged 2 times in line mode. Depth scale is shown in the right lower corner. CN: thin column; CK: thick column; TM: transversal mitochondria. Both white scale bars on the picture correspond to 5 µm.

RPmt is targeted to the mitochondrial matrix by the sequence encoding the N-terminal 12 amino acids presequence of subunit IV of cytochrome c oxidase. Confocal imaging revealed that RPmt was colocalised with TMRM, a mitochondrion-selective membrane potential indicator (fig. 1A, B, C). RPmt stained intermyofibrillar and subsarcolemmal mitochondria, with little if any cytosolic contamination (fig. 1B). 3D reconstruction (fig. 1D) of the intermyofibrillar mitochondrial network, as highlighted by RPmt, unveiled a very sharp pattern with paired slender mitochondria transversally aligned on both sides of the Z line, and thin and a few thick longitudinal mitochondrial columns, as previously described at electron microscopy resolution [12]. The results show that my experimental conditions allow a highly precise targeting of the protein.

### Spontaneous bursts of fluorescence reflect an increase of superoxide

**Figure 2:**
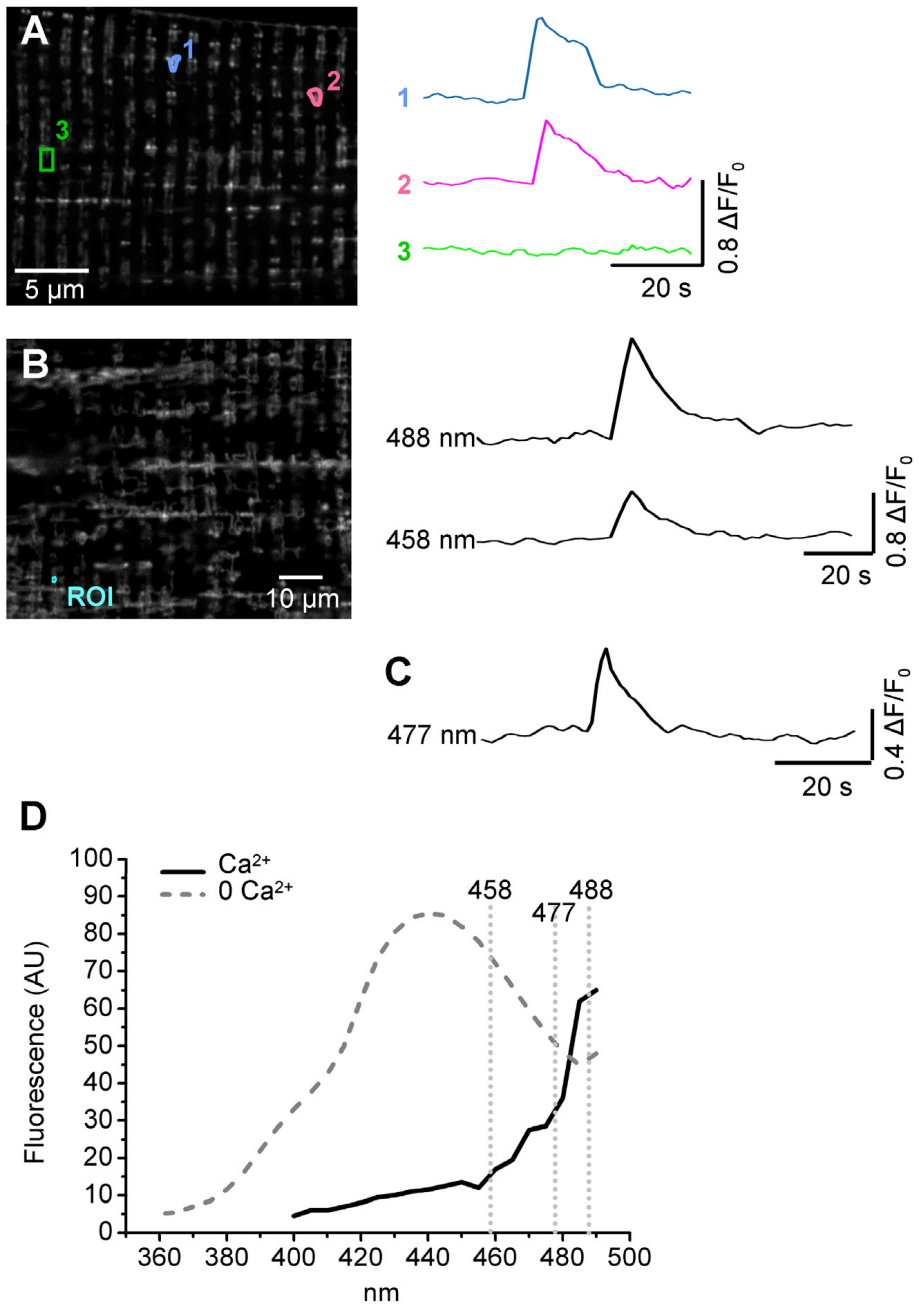
Spectral properties of the spontaneous bursts of fluorescence. **A** Left, confocal image (x,y) of fluorescence of a fiber transfected with RPmt. RPmt was excited at 488 nm. Right, time course of changes in normalized RPmt fluorescence within the Regions Of Interest (ROI) 1, 2 and 3. **B** Left, confocal image (x,y) of fluorescence of a skeletal muscle fiber transfected with RPmt. Right, time course of changes in normalized fluorescence of RPmt, stimulated either at 488 nm or at 458 nm, within the ROI. **C** This trace, recorded in a different cell, shows the time course of changes in normalised fluorescence of RPmt, stimulated at 477 nm, which is close to the isoemissive point for calcium. **D** Excitation spectrum of RPmt recorded in transfected skeletal muscle fibers. Excitation wavelength increases by 10 nm steps from 360 to 490 nm. Emission was measured at 520–560 nm. Fibers were incubated in tyrode with 100 µM EGTA AM (0 Ca^2+^) or 20 µM ionomycine (Ca^2+^).

Repeated xy confocal imaging of RPmt revealed an exciting phenomenon: ∼20 s-long bursts of fluorescence with an average ΔF/F_0_ of 0.93±0.02 (n = 1328 flashes from 34 cells) (see fig. 2A and movie S1 in supplemental material).

Due to the nature of RPmt, a calcium sensor, these events were first interpreted as transient increases of mitochondrial matrix calcium concentration. However, spectral properties of these events presented discrepancies with the calcium-dependence of the excitation spectrum provided by Nagai et al. [11] and of the spectrum recorded here in transfected skeletal muscle fibers (fig. 2D). Increases in RPmt fluorescence were observed simultaneously at the excitation wavelengths of 488 and 458 nm (fig. 2B) whereas calcium-induced changes in fluorescence should have opposite directions at these wavelengths. In addition, increases in RPmt fluorescence were also recorded at 477 nm, which is very close to the isoemissive point for calcium (fig. 2C).

**Figure 3:**
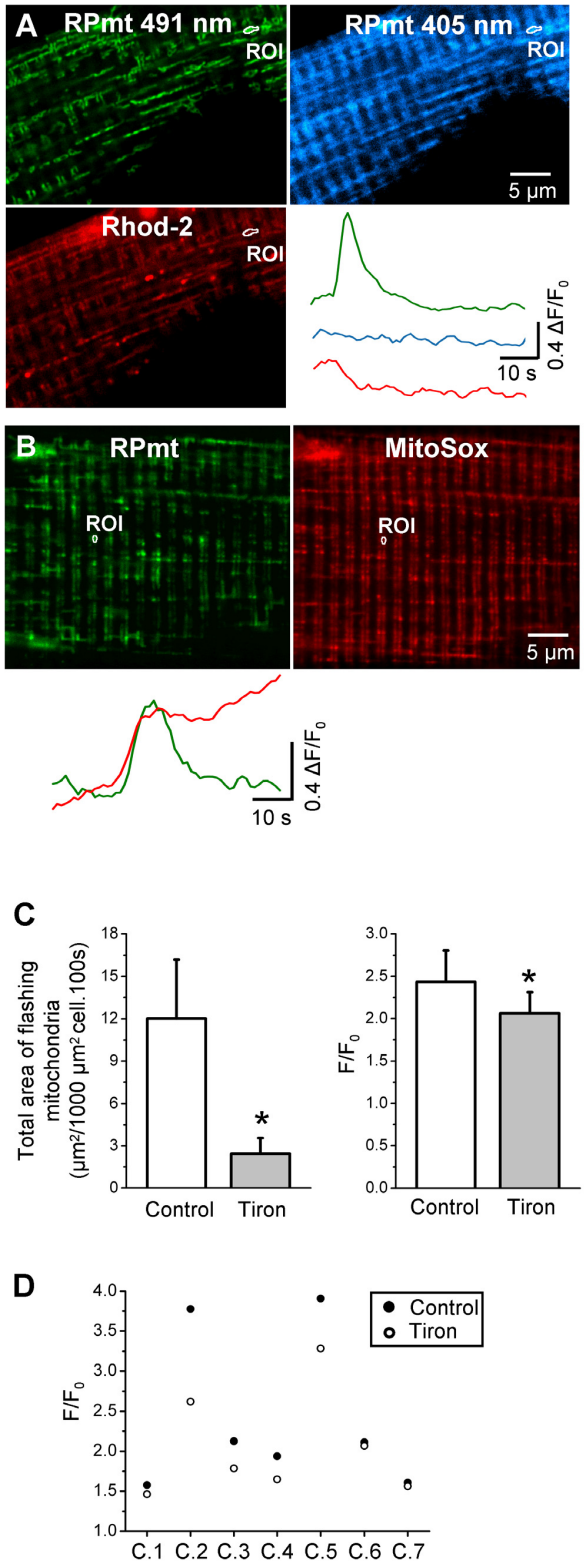
Spontaneous bursts of fluorescence reflect increases of superoxide levels in mitochondria. **A** Confocal images of a fiber transfected with RPmt (excitation wavelength 491 nm and 405 nm) and loaded with Rhod-2. Traces represent the time course of changes in normalised RPmt fluorescence (green: 491 nm excitation; blue: 405 nm) or Rhod-2 fluorescence (red) in the ROI. **B** Confocal images of a fiber transfected with RPmt and loaded with MitoSox. Traces represent the time course of change in normalized RPmt (green) or MitoSox (red) fluorescence in the ROI. Results were reproduced on 6 cells. **C** Total area of flashing mitochondria per 1000 µm^2^ cell per 100 s (left) and average amplitude of flashes (right) in 7 cells under control conditions and after 20 min of treatment with 15 mM tiron (*: p<0.05 for tiron vs control, paired Student's t test). **D** Average amplitude of flashes for each of the 7 cells (C.1–C.7) included in C, under control condition and after 20 min of treatment with 15 mM tiron.

To further explore these discrepancies, I monitored changes in fluorescence intensity of RPmt using the 405 and 491 nm laser lines as excitation wavelengths while simultaneously recording changes in mitochondrial [Ca^2+^] using the fluorescent dye Rhod-2. Spontaneous bursts of RPmt fluorescence were observed with the 491 nm excitation wavelength but not with the 405 nm (fig. 3A). This was not compatible with a calcium-induced change in fluorescence since, in this case, a strong decrease of fluorescence at 405 nm should accompany an increase of fluorescence at 491 nm (fig. 2D). Moreover, the simultaneous measurement of Rhod-2 fluorescence showed a decrease of mitochondrial [Ca^2+^] concomitant with the increase of RPmt fluorescence, strongly arguing against the possibility that the events reflect calcium movements.

During the course of this work, Wang et al. [10] showed that the fluorescent protein cpYFP of the ratiometric pericam can specifically sense superoxide. The authors created a new superoxide sensor, called mt-cpYFP by removing the calcium sensing part (calmodulin (nt 886–1323) and M13 (nt 49–126)) from the RPmt. Hence, in addition to sensing calcium, RPmt is also sensing superoxide, most likeky with the same properties than mt-cpYFP. Interestingly, mt-cpYFP spectral properties are compatible with my records using different excitation wavelengths. Hence, the events could reflect transient increases of superoxide level, detected by the cpYFP part of the RPmt, rather than transient increases in calcium concentration. Several lines of evidences confirmed this hypothesis. First, the records showed simultaneous increases in the fluorescence intensity of MitoSox, a mitochondrial specific synthetic probe selective for superoxide, and RPmt fluorescence (fig. 3B). The continuous increase in the MitoSox fluorescence intensity during the time course of the experiment was undoubtedly due to non-reversibility of the fluorescence change in this dye. These results were reproduced on 6 other cells. Second, application of 15 mM tiron, a superoxide scavenger, to the fiber decreased the frequency of events from 12±4.2 to 2.4±1.1 µm^2/^1000 µm^2^ cell.100 s (n = 7 cells), and the average events amplitude F/F_0_ from 2.43±0.37 to 2.06±0.25 (fig. 3C).

**Figure 4:**
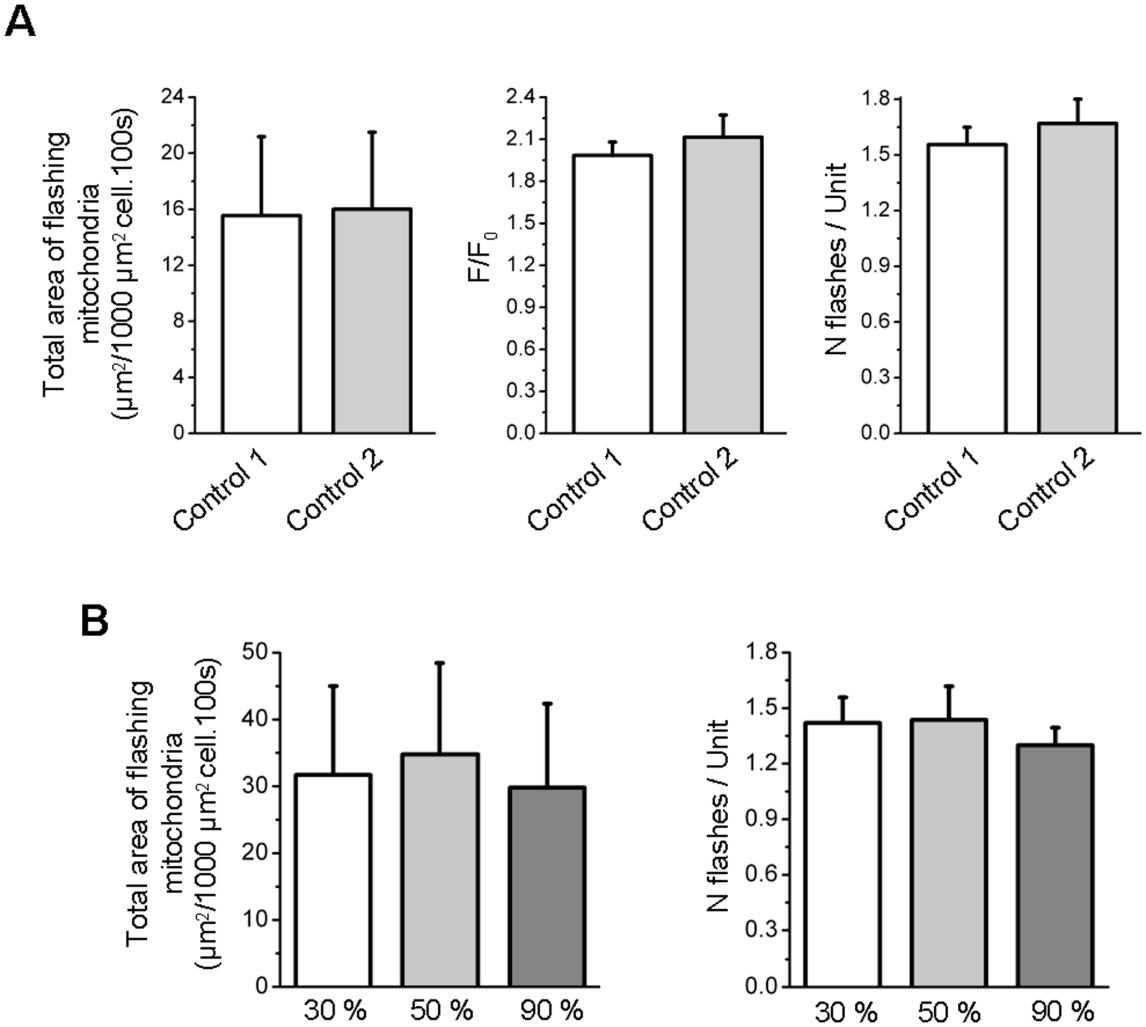
Superoxide flashes are not induced by photostimulation. **A** Total area of flashing mitochondria per 1000 µm^2^ cell per 100 s (left), average amplitude of flashes (middle) and number of flashes per active units (right) in 7 cells during two successive time series of 3 min (CN 1 and CN 2). Paired Student's t tests have been performed. **B** Total area of flashing mitochondria per 1000 µm^2^ cell per 100 s (left, n = 8), and number of flashes per active units (right, n = 5) during 3 successive time series of 100 s, with increasing laser intensity. Friedman ANOVA has been performed. Note: Cri in the detection macro was set to 1.5 in this series of experiments, as the signal over noise ratio was greatly reduced for the data obtained with 30% laser transmission. This reduced the resolution of the detection algorithm, and false positives had to be removed manually.

For the sake of nomenclature uniformity, the spontaneous bursts of RPmt fluorescence were named “flashes”, following the terminology of Wang et al. [10]. In order to avoid calcium contamination in the superoxide signals, RPmt was then systematically excited at 477 nm. Superoxide flashes occurred randomly in time. Their size presented important variability. In order to encompass both their number and their size, flashes frequency is reported in this paper in total area of flashing mitochondria per area of cell per unit of time (µm^2/^1000 µm^2^ cell.100 s). Average flash frequency was 18±2.4 (n = 42 cells), but varied substantially between different fibers. Hence, to study the effect of a given condition on the properties of the events, records were performed in a same fiber under control condition and after treatment, and paired statistical tests were used. In order to discard any effect of laser exposure on events properties, which may interfere with the treatment, two 3 min time series were successively recorded on the same fiber. The frequency and properties of the flashes during the first and the second time series were similar (n = 7, see fig. 4A). This shows that, at least under the current experimental conditions, superoxide flashes properties are not modified by putative damaging effects induced by long lasting laser exposure.

Bursts of mitochondrial ROS have been shown in cardiac cells following artificial ROS production by photoactivation of the mitochondrion-selective membrane potential indicators TMRM and TMRE [13], [14]. In the present work, superoxide flashes occured also in cells transfected with RPmt but devoid of any synthetic dye (TMRM or other). In order to rule out the possibility that superoxide flashes result from artificial ROS production, for instance following excitation of RPmt, the following protocol was performed: muscle fibers transfected with RPmt were successively exposed to increasing intensities of a 477 nm laser line (laser transmission: 30 %, 50 %, 90 %). Neither the frequency nor the properties of the flashes were affected by the increase in laser exposure intensity (fig. 4B). This result demonstrates that the superoxide flashes recorded in this study are spontaneous cellular events and not artifacts induced by recording conditions.

### Subcellular localization of superoxide flashes

**Figure 5:**
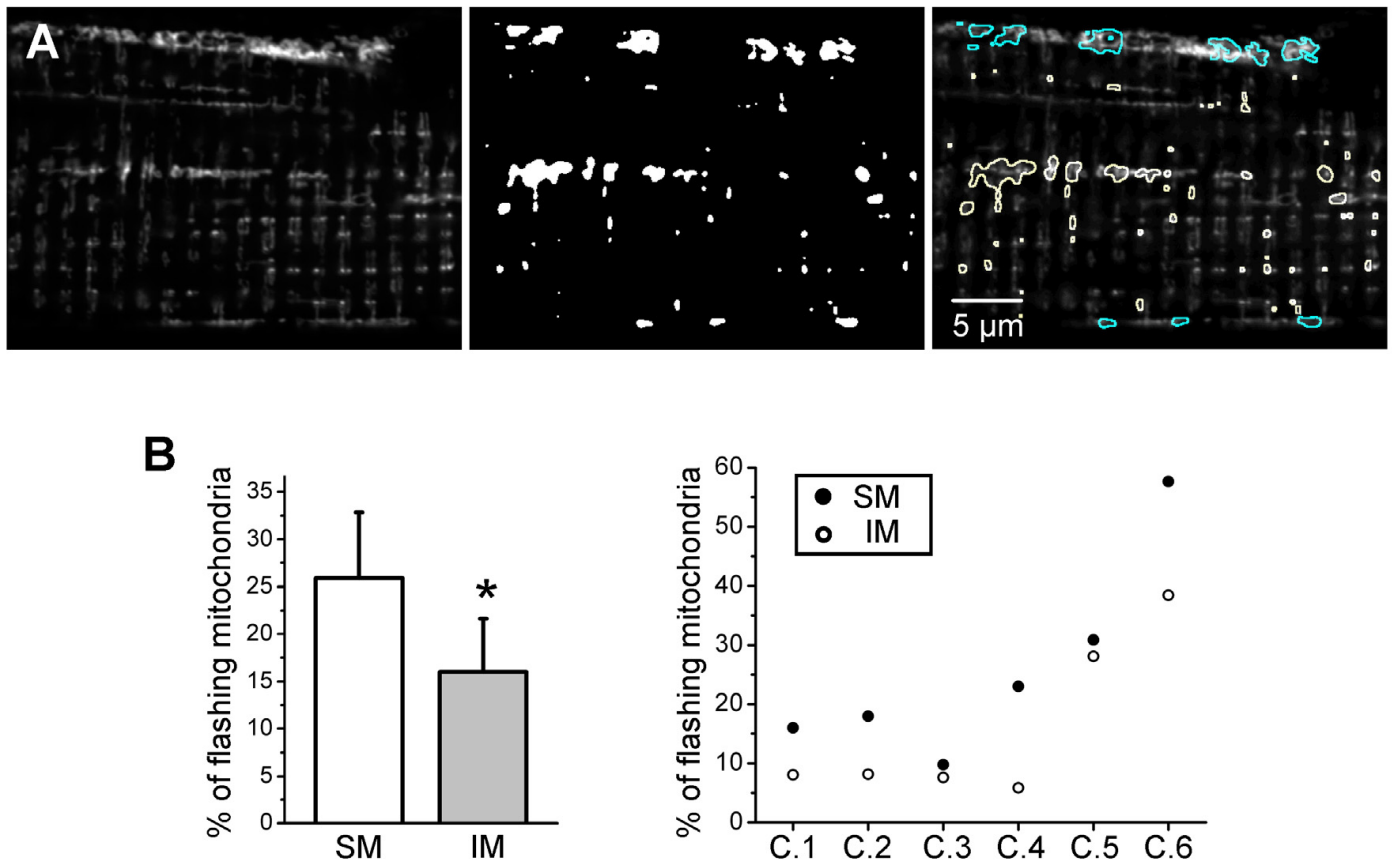
Subcellular distribution of superoxide flashes. **A** Left, confocal image (x,y) of fluorescence of a fiber transfected with RPmt. Middle, binary mask of all flashes detected in the 3 min time series. Right, overlay showing in blue the flashes arising in subsarcolemmal mitochondria and in yellow the flashes in intermyofibrillar mitochondria. **B** Left, percentage of flashing mitochondria among subsarcolemmal or intermyofibrillar mitochondria during a 3 min time series (n = 6, *: p<0.05 for SM vs IM, paired Wilcoxon Signed Rank test). SM: subsarcolemmal mitochondria; IM: intermyofibrillar mitochondria. Right, percentage of flashing mitochondria among subsarcolemmal or intermyofibrillar mitochondria during a 3 min time series for each of the 6 cells included in the left panel.

Superoxide flashes were observed in subsarcolemmal mitochondria, as well as in intermyofibrillar mitochondria (fig. 5A). Their relative distribution between these two subcellular compartments was not random, as they were more frequent in subsarcolemmal mitochondria than in intermyofibrillar ones (25.9%±7 of flashing mitochondria among the subsarcolemmal ones vs 16%±5.6 among the intermyofibrillar ones, n = 6 cells, see fig. 5B).

**Figure 6:**
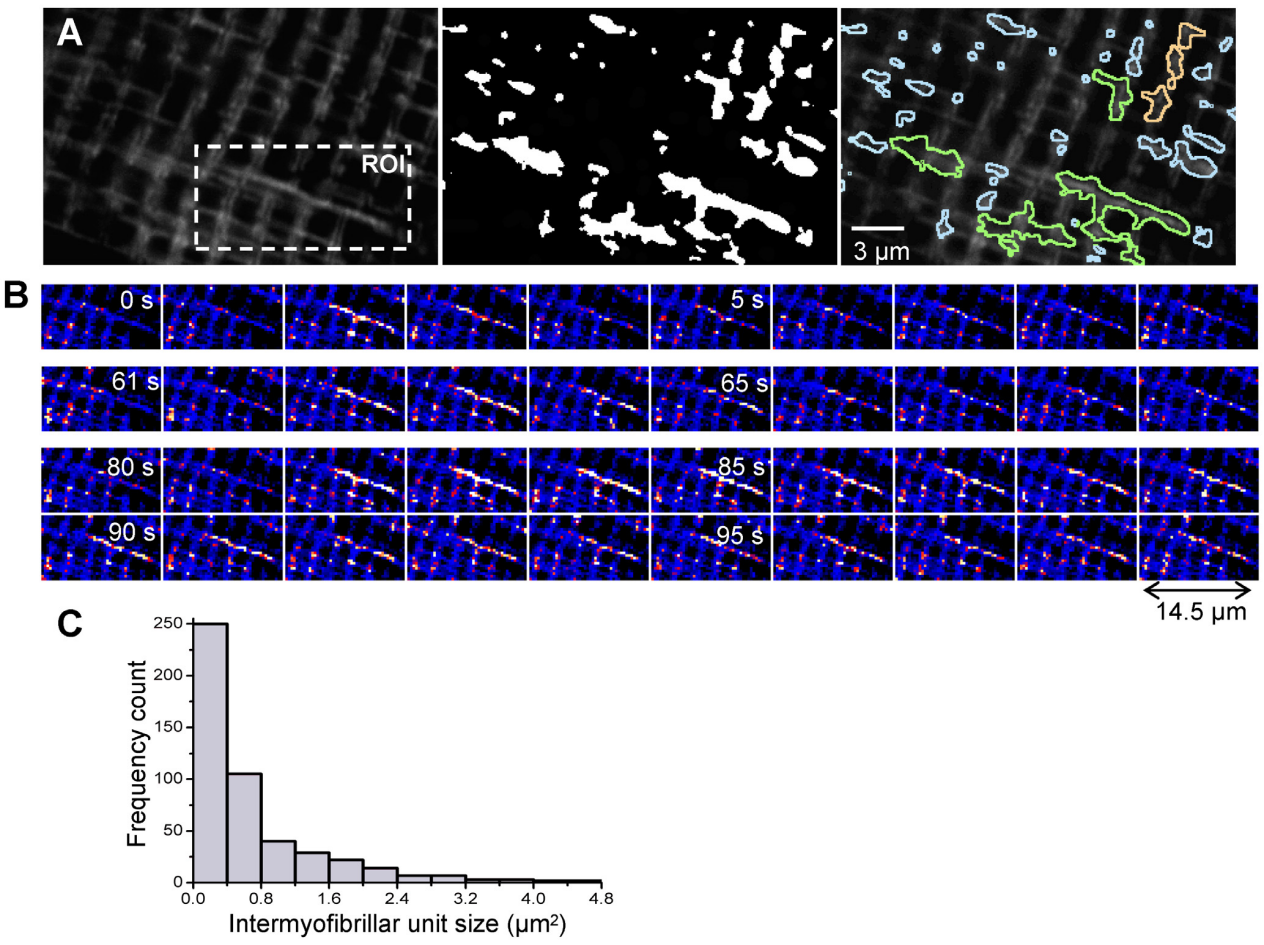
Shape and size of intermyofibrillar flashes. **A** Left, confocal image (x,y) of the fluorescence of a fiber transfected with RPmt. Middle, binary mask of all flashes detected in the 3 min time series. Right, overlay, showing in blue the flashes involving probably a single mitochondrion, in orange the flashes involving a pair of longitudinal mitochondria, and in green the flashes involving larger clusters of longitudinal and transversal mitochondria. The array of mitochondria involved in the same flash is called an active unit. **B** Imaging of an active unit involving a cluster of mitochondria, at 1 s intervals. The image is shown in pseudo-colored scale. **C** Histogram of distribution of active units sizes with x-axis ranging from 0 to 4.8 µm^2^. The histogram corresponds to 490 flashes recorded on 7 cells. Binning interval was 0.4 µm^2^. Point shaped units have an area of approximately 0.088–0.8 µm^2^, and column shaped units of 0.8–1.6 µm^2^. Unit constituted of a pair of longitudinal mitochondria measure roughly 1.6–2.4 µm^2^. Larger size-units (up to 18 µm^2^) are clusters of transversal and longitudinal mitochondria.

Events recorded in intermyofibrillar mitochondria presented different patterns (fig. 6A). Some seemed to involve a single mitochondrion, point shaped or column-shaped, longitudinal or transversal. Point shaped events had an area of approximately 0.088–0.8 µm^2^, while column shaped events were roughly 0.8–1.6 µm^2^. Others (1.6–2.4 µm^2^) comprised a pair of longitudinal mitochondria. Finally, a third class of events encompassed large clusters of mitochondria and involved thick longitudinal columns. The histogram of distribution of flashes size (fig. 6C) revealed an inverse correlation between size and frequency, the point-shaped events being the most frequent (n = 490 events on 7 cells). The large clusters, which areas could vary from 2.4 up to 18 µm^2^, were less common than the other patterns, and not found in every cell. It should however be kept in mind that, as shown in fig. 1D, mitochondrial network encompasses several confocal planes. Hence flashes size could be underestimated here, as measures are performed in a single confocal plan.

Flashes of any size occurred amidst a background of quiescent mitochondria, suggesting that flashes do not simply propagate to adjacent mitochondria under resting conditions. The spatial distribution of flashes was not random, but linked to the morphology of mitochondria. Hence, thick longitudinal columns were often involved in large clusters (fig. 6A). On the other hand, point shaped and column shaped events were found in longitudinal thin column, or transversal paired slender mitochondria.

Even the largest cluster-sized flashes presented a striking homogeneity in terms of increase of fluorescence intensity (fig. 6B), suggesting either a continuity of mitochondrial lumen in the entire cluster, or the existence of superoxide-permeant junctions between the mitochondria constituting a cluster. Furthermore, the same cluster could flash several times during a 3 min record (fig. 6B), the shape of the successive flashes being identical. It should be stressed that the possibility of changes in the flash shape due to mitochondrial movements should be discarded because mitochondria are essentially immotile in skeletal muscle at the records time scale, mostly due to their strong attachment to calcium release units [15].

Overall, results show the existence of intermyofibrillar mitochondrial units of variable size, characterized by their superoxide production. These units have well-defined boundaries and are stable in time. ‘Active’ units are surrounded by quiescent ones; this shows that superoxide does not diffuse randomly to adjacent mitochondria.

### Flashes are metabolism-linked events

**Figure 7:**
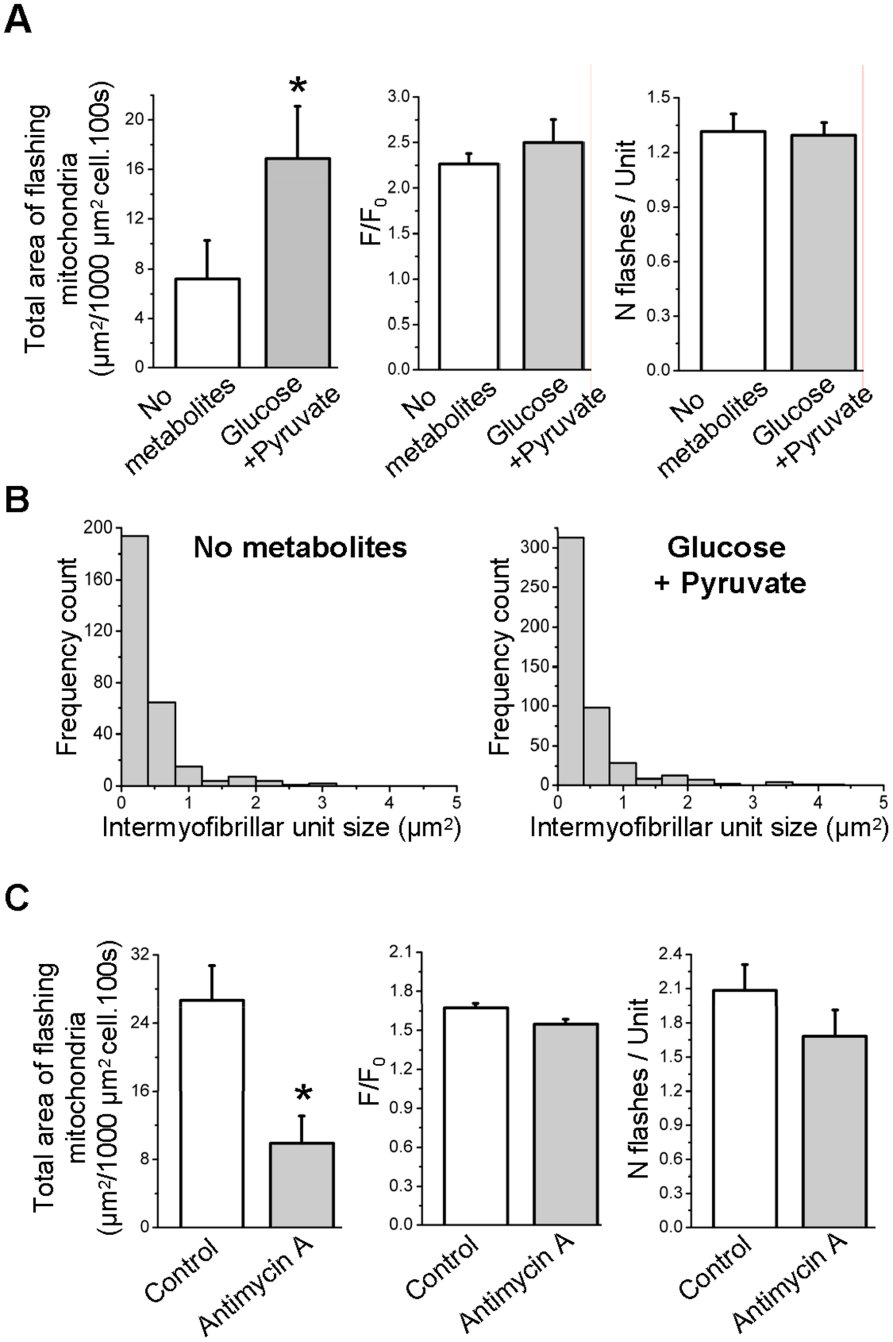
Superoxide flashes are linked to metabolism and involve the electron transport chain. **A** Total area of flashing mitochondria per 1000 µm^2^ cell per 100 s (left, n = 12 cells), average amplitude of flashes (middle, n = 10) and number of flashes per active unit (right, n = 10 cells) in cells incubated in a tyrode solution devoid of metabolites, and after application of a tyrode solution containing 10 mM glucose and 5 mM pyruvate. (*: p<0.05 for “No metabolites” vs “Glucose + Pyruvate”, paired Student's t test) **B** Histogram of distribution of active units sizes in 9 cells incubated in a tyrode solution devoid of metabolites, and after application of a tyrode solution containing 10 mM glucose and 5 mM pyruvate. X-axis ranges from 0 to 5 µm, as there are very few counts beyond this value. Binning interval is 0.4 µm^2^. **C** Total area of flashing mitochondria per 1000 µm^2^ cell per 100 s (left), average amplitude of flashes (middle) and number of flashes per active unit (right) in 5 cells under control conditions, and after a treatment with 2.5 µM antimycin A. (*: p<0.05 for control vs antimycin A, paired Wilcoxon Signed Rank test).

As flashes seem to be ubiquitous events in different cell types [10] and, in a same cell, among different types of mitochondria (fig. 5), they might be linked to the first role of the mitochondrion, the regulation of cellular metabolism. Indeed, flash frequency was low (7.2±3.1 µm^2/^1000 µm^2^ cell.100 s, n = 12) when fibers were incubated in a Tyrode solution devoid of metabolites and increased to 16.9±4.2 (n = 12) upon application of 10 mM glucose and 5 mM pyruvate (fig. 7A). Surprisingly, neither the flashes amplitude nor the number of flashes per mitochondrial unit (fig. 7A) were affected. Furthermore, the distribution of intermyofibrillar unit size was identical in fibers incubated with Tyrode devoid of or containing metabolites (fig. 7B), whereas the number of flashing units was higher in fibers incubated in Tyrode containing glucose and pyruvate. These results suggest that mitochondria work as restricted units of production of superoxide, and that increase in cell superoxide production is due to a recruitment of supplementary units rather than an increase of superoxide production per unit.

Inhibition of the electron transport chain with 2.5 µM antimycin A, an inhibitor of complex III, decreased flashes frequency from 26.7±4.1 to 9.9±3.2 µm^2^/1000 µm^2^ cell.100 s (n = 5, fig. 7C), without affecting the flashes amplitude or the number of flashes per unit, demonstrating an involvement of the ETC in the flashes production. Overall, these results show that flashes are metabolic –linked events involving the ETC.

### Superoxide flashes are accompanied by mitochondrial depolarization

**Figure 8:**
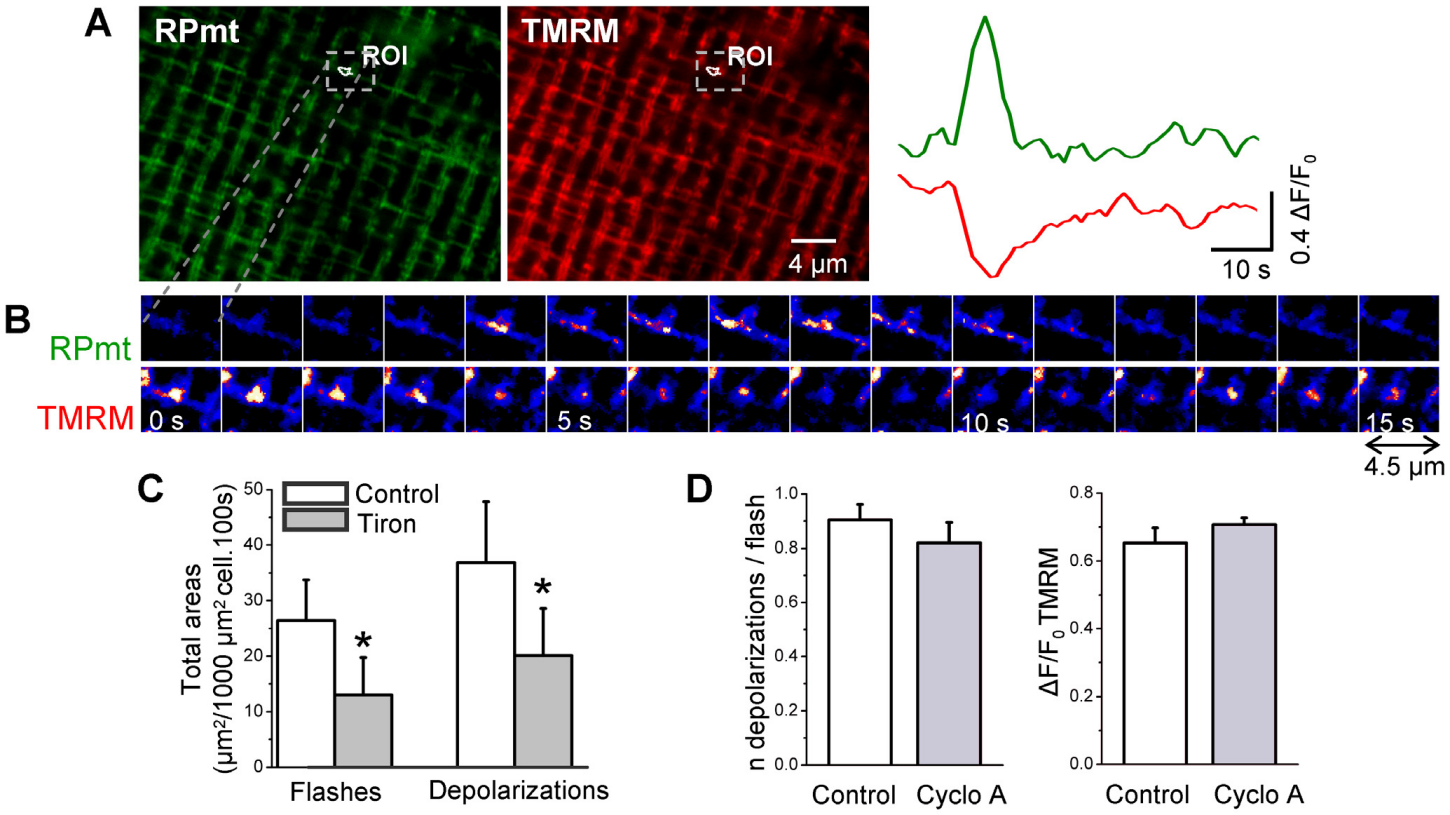
Superoxide flashes induce mitochondrial depolarizations, independently from the activity of the permeability transition pore. **A** Left, confocal images (x,y) of fluorescence of a skeletal muscle fiber transfected with RPmt and loaded with TMRM. Right, time course of changes in normalised RPmt (green) or TMRM (red) fluorescence in ROI. **B** Enlarged views showing imaging of RPmt and TMRM fluorescence at 1 s interval. The images are shown in pseudo-coloured scale. Note the spatial and temporal colocalization of the increase in RPmt and decrease in TMRM fluorescence. **C** Total area of mitochondria presenting a flash or a depolarization per 1000 µm^2^ cell per 100 s, in 5 cells under control conditions and after 20 min of treatment with 15 mM tiron (*: p<0.05 for control vs tiron, paired Wilcoxon Signed Rank test). **D** Number of depolarizations concomitant to superoxide flashes (left), and depolarization amplitude (right) in 6 cells under control conditions and after a 20 min treatment with 5 µM cyclosporin A (cyclo A). Paired Wilcoxon Signed Rank tests have been performed.

86% of superoxide flashes were accompanied by a mitochondrial depolarization, as shown by the simultaneous records of RPmt fluorescence and TMRM (fig. 8A, B). These depolarizations occurred exactly at the same spatial location than the flashes, which reinforces the hypothesis of separated mitochondrial units for the production of superoxide. The depolarizations had a mean amplitude ΔF/F_0_ of 0.57±0.01 (n = 391 flashes from 11 cells), the minimal amplitude being 0.11. Hence, the 14% of flashes that appeared to be devoid of depolarization were probably due to a lack of resolution of the technique rather than a lack of depolarization. These depolarizations did not have systematically the same kinetics as the flashes. They could be square-shaped or long lasting (with no recovery during the course of the record). In rare cases (2–3% of the flashes), the flash-linked depolarization was preceded by a transient mitochondrial hyperpolarization. Finally, some spontaneous depolarizations were devoid of flashes.

Wang et al. [10] suggested that mitochondrial depolarization causes the superoxide flash. In order to check this hypothesis in my model, I applied tiron to the cells, and measured flash and depolarization frequency. My results show that depolarizations frequency is higher than flashes frequency (36.9±11 µm^2^/1000 µm^2^ cell.100 s versus 26.4±7.4 respectively, n = 5), which is expected since some depolarizations are independent of flashes. As described above, flash frequency decreased with tiron from 26.4±7.4 µm^2^/1000 µm^2^ cell.100 s to 13±6.8 (n = 5). Surprisingly, depolarization frequency also decreased, to the same extent (36.9±11 µm^2^/1000 µm^2^ cell.100 s to 20.2±8.5), arguing that the depolarization is caused by the superoxide flash, rather than the opposite (fig. 8C).

I next tried to characterize the protein(s) involved in the mitochondrial depolarization (fig. 8A) and concomitant calcium exit (fig. 3A) accompanying the flash. Several studies suggested that ROS induce opening of the PtP, causing a mitochondrial depolarization [13], [16], [17]. However, in the present conditions, blocking the PtP with 5 µM cyclosporin A neither affected the frequency nor the amplitude of the ROS-induced depolarization. Lower concentration of cyclosporin A (0.2 and 1 µM) were also found ineffective (results not shown). This strongly suggests that the PtP is not involved in the flash-induced depolarization (fig. 8D).

### Tiron treatment affects the kinetics of the flashes

**Figure 9:**
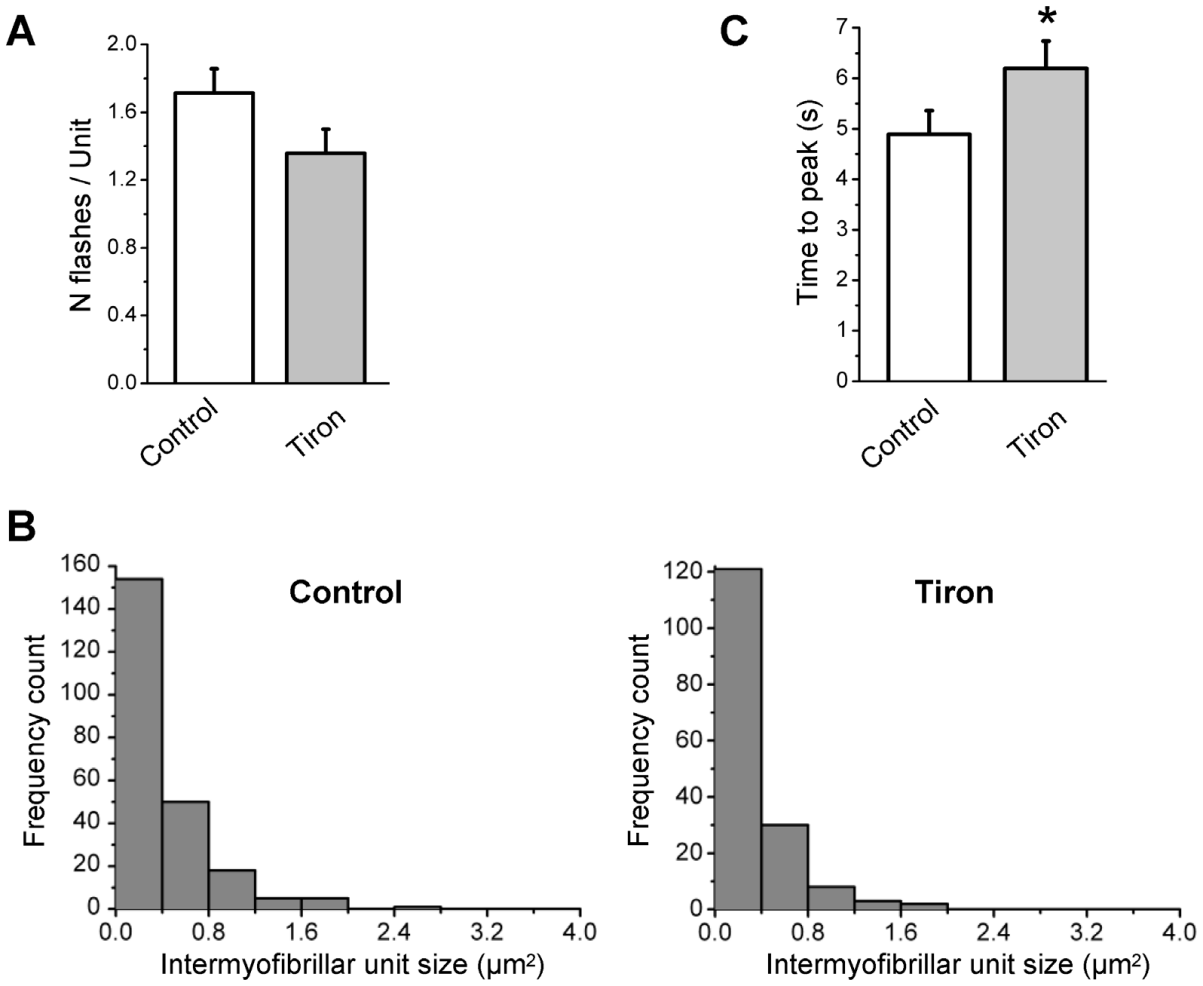
Superoxide scavenger decreases the number of active units and extends the flashes rising phase. **A** Number of flashes per active unit in 7 cells under control conditions and after 20 min of treatment with 15 mM tiron. **B** Histogram of distribution of active units sizes in 7 cells under control conditions and after 20 min of treatment with 15 mM tiron. **C** Time to peak (s) of the flashes in 7 cells under control conditions and after 20 min of treatment with 15 mM tiron (*: p<0.05 for control vs tiron, paired Student's t test).

As discussed before, tiron treatment decreased the amplitude of the flashes, as well as the flashes frequency. Conversely, fig. 9 shows that tiron did not significantly affect the number of flashes per unit (fig. 9A) or the distribution of intermyofibrillar unit sizes (fig. 9B). However, time to peak was significantly increased upon tiron application (fig. 9C: 4.89±0.47 s under control conditions vs 6.2±0.54 s with tiron, n = 7). This strongly suggests that superoxide regulates its own production. Interestingly, neither the τ decay (τ = 8.23±1.03 s^−1^ control vs τ = 10.23±1.79 s^−1^ tiron, n = 5) nor the full width at half magnitude (8.06±0.95 s control vs 6.98±0.68 s tiron, n = 5) were affected.

### Mechanisms underlying flashes genesis

**Figure 10:**
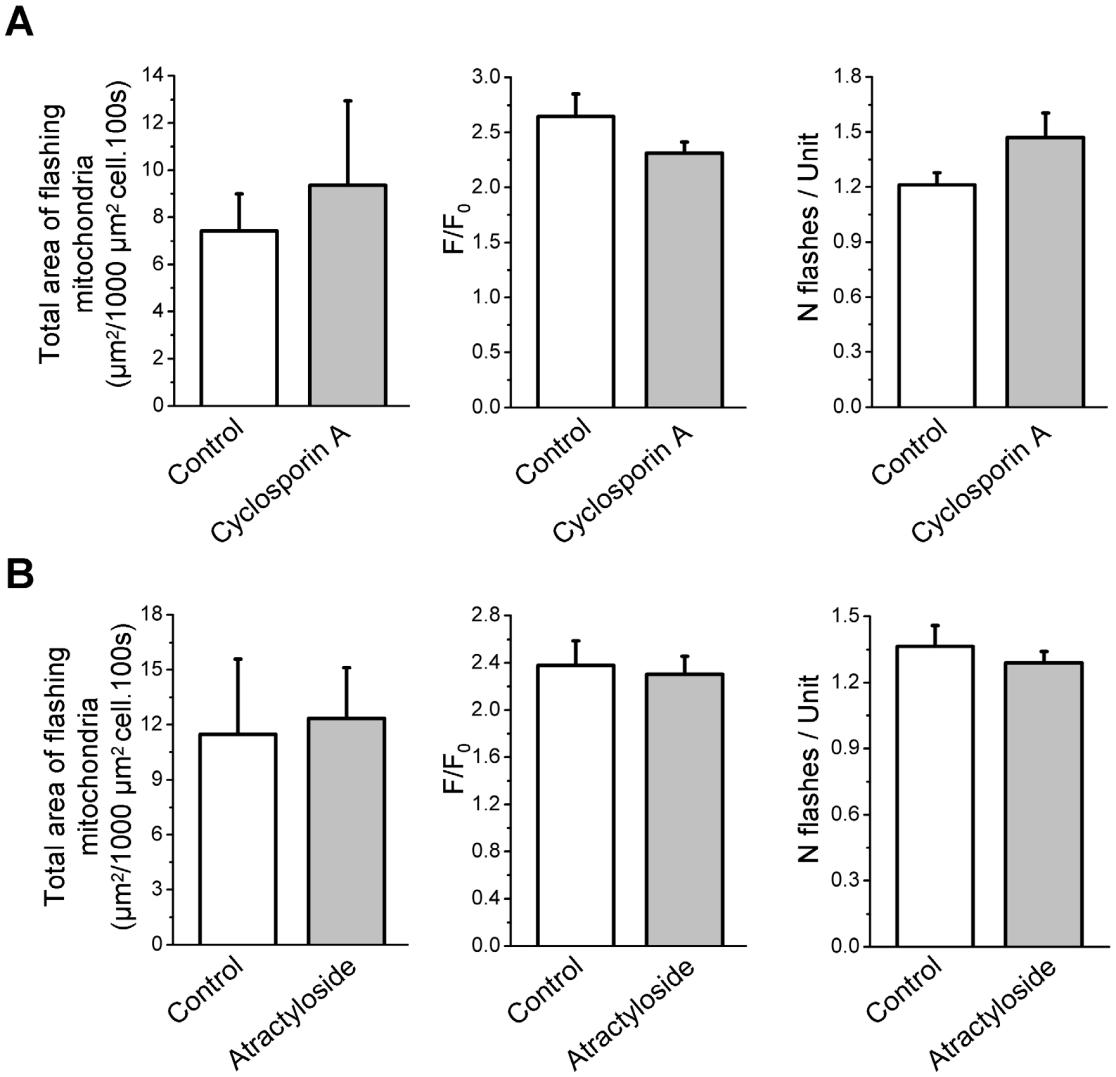
Superoxide flashes are not induced by openings of the permeability transition pore. **A** Total area of flashing mitochondria per 1000 µm^2^ cell per 100 s (left), average amplitude of flashes (middle) and number of flashes per active unit (right) in 5 cells under control conditions and after a 20 min treatment with 5 µM cyclosporin A. Paired Wilcoxon Signed Rank tests have been performed. **B** Total area of flashing mitochondria per 1000 µm^2^ cell per 100 s (left), average amplitude of flashes (middle) and number of flashes per active unit (right) in 8 cells under control conditions and after a 20 min treatment with 50 µM atractyloside. Paired Student's t tests have been performed.

Stochastic openings of the PtP have been shown to trigger superoxide flashes genesis in cardiac myocytes [10]. Hence, to test the hypothesis that PtP may be involved in flashes production in skeletal muscle, cyclosporin A (a PtP inhibitor) and atractyloside (a PtP activator) were applied. Neither inhibition of PtP with 5 µM cyclosporin A (fig. 10A) nor activation of PtP with 50 µM atractyloside (fig. 10B) affected flashes frequency or properties, suggesting that another mechanism is involved in superoxide flashes production in skeletal muscle fibers.

**Figure 11:**
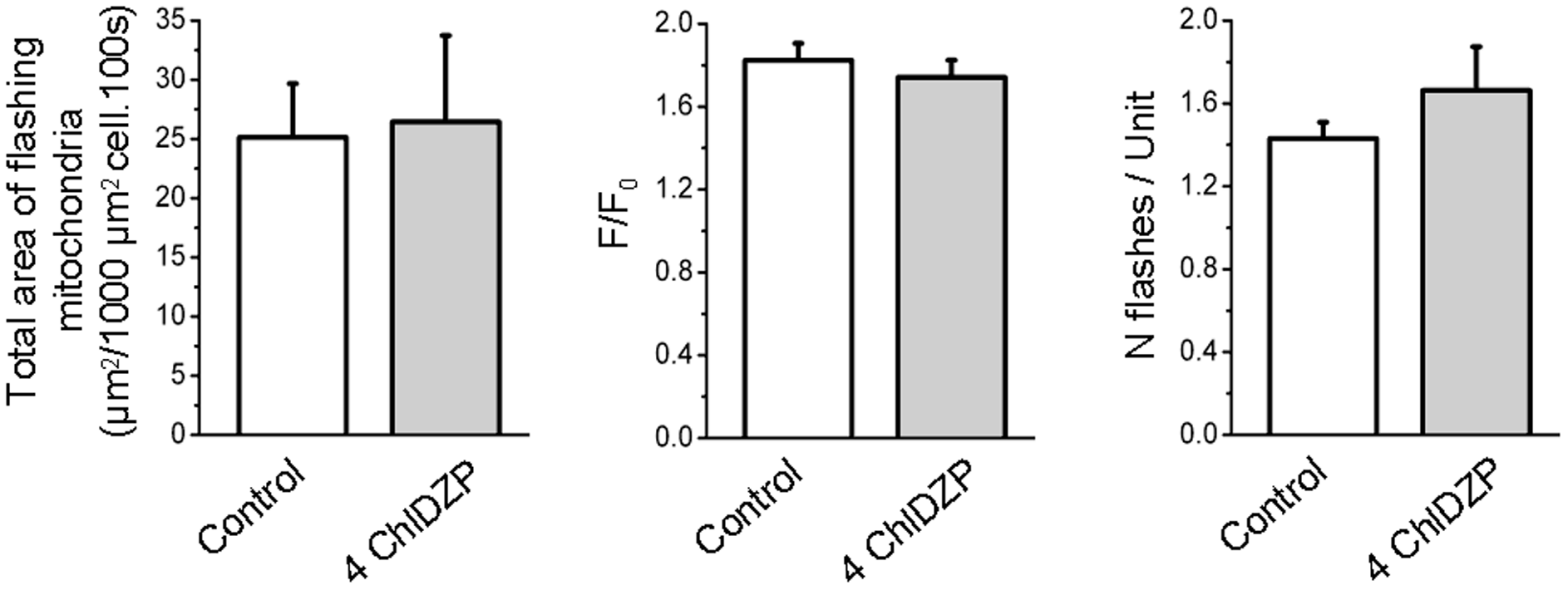
Anion channel inhibitor does not affect superoxide flashes properties. Total area of flashing mitochondria per 1000 µm^2^ cell per 100 s (left), average amplitude of flashes (middle) and number of flashes per active unit (right) in 8 cells under control conditions and after treatment with 40 µM 4′-chlorodiazepam (4-ChlDZP). Paired Student's t tests have been performed.

Another mitochondrial channel could be involved in the production of superoxide flashes: IMAC. IMAC mediated mitochondrial propagation of ROS events has been shown in cardiac myocytes following local perturbation of a few mitochondria by phototoxicity [14]. Therefore, the effects of 4′ chlorodiazepam (4-ChlDZP), a reported inhibitor of IMAC, were tested. Application of 40 µM 4-ChlDZP did not affect flashes frequency nor properties (fig. 11). Also, in 2 cells transfected with RPmt and loaded with TMRM, 4-ChlDZP application failed to inhibit mitochondrial depolarization induced by superoxide flashes (result not shown). These results show that IMAC is neither involved in physiological superoxide flashes production nor in flashes-induced mitochondrial depolarizations in skeletal muscle.

## Discussion

In the present work, RPmt was used as a superoxide biosensor targeted to mitochondria of adult skeletal muscle fibers. Repeated xy confocal imaging of RPmt revealed superoxide flashes with an approximate duration of 20 s, and an average amplitude ΔF/F_0_ of 0.93±0.02. Mitochondrial superoxide flashes appear as discrete events arising amidst a quiescent background. They involve clusters of active mitochondria, or active units, whose shape relies heavily on the organization of the mitochondrial network. Flashes are not a consequence of damaging processes, such as pathological phenomenon, laser exposure, oxidative stress or metabolic stress. Rather, they seem to be physiological events, linked to cellular metabolism. In their turn, superoxide flashes modulate mitochondrial function, as they cause mitochondrial depolarizations.

Superoxide flashes genesis is shown to involve ETC activity as ETC blockade by antimycin A dramatically decreases superoxide flashes frequency. Interestingly, no significant differences were found in basal mitochondrial level of superoxide (F0) in cells under control and antimycin conditions (result not shown). Antimycin A is commonly seen as an enhancer of ROS production through accumulation of ubisemiquinone at complex III. However, discrepancies have been described in the literature. For instance, antimycin A has been reported to decrease superoxide flashes frequency in cardiac myocytes [10], or to reduce glucose deprivation-induced ROS production in cerebellar granule neurons [18]. Discrepancies may arise from different experimental conditions or probes (DCF, Amplex Red indirectly measuring superoxide through its by-product hydrogen peroxide or hydroxyl radical vs hydroethidine, mt cpYFP which directly measure superoxide levels). Also, mitochondrial matrix levels of superoxide may be differently regulated from cytosolic ones. Indeed, Kudin et al. [19] showed that antimycin A induces an increase of superoxide levels in isolated mitochondria from rat brain and skeletal muscle, this increase taking place outside the mitochondrial matrix.

Superoxide flashes are not randomly distributed among the subpopulations of mitochondria. They were found to be in higher frequency in subsarcolemmal mitochondria than in intermyofibrillar ones. Metabolic differences between these two populations of mitochondria were observed in permeabilized muscle fibers from mouse quadriceps, rat soleus and gastrocnemius [20], [21]. By measuring FAD/NADH autofluorescence, which reflects the activity of the ETC, the authors showed that subsarcolemmal mitochondria are in a more oxidative state than intermyofibrillar ones. The higher flashes frequency observed in this work in subsarcolemmal mitochondria could be well explained by a higher ETC activity.

Superoxide flashes in intermyofibrillar mitochondria involve active mitochondrial clusters adjacent to quiescent ones. These active units have very well defined limits and are stable in time, as superoxide flashes can be detected several times at the same place during a 3 min record. The distribution and shape of active units in skeletal muscle relies on the mitochondrial organization, especially on the presence of thin and thick columns, as described in [12]. For instance, large units (clusters) systematically involve longitudinal thick columns. Unit size varies from 0.1 µm^2^ up to 18 µm^2^. The fact that mitochondrial depolarizations, as detected with TMRM, occur exactly at the same place as superoxide flashes shows that mitochondrial clusters are composed of electrically connected mitochondria. This connection could be due to a continuous lumen, or to conductive junctions between mitochondria. Junctions of high electric conductance have been described by Amchenkova et al. [22] in rat cardiomyocytes. These junctions define mitochondrial clusters by connecting together several mitochondria. Such networks are common and have been described in several cellular types [23]. These mitochondrial clusters may constitute subcellular microdomains for fine tune of energy production and delivery, and organisation of antioxidant defense, as discussed by [23].

Superoxide flashes are accompanied by transient depolarization of mitochondria. Spontaneous transient depolarizations of mitochondrial membrane potential, sometimes called mitochondrial flickers [24], are a wide spread phenomenon. They have been for instance observed in neuroblastoma cells [25], cardiomyocytes [10], [13], [24], [26], smooth muscle cells [17], [27], vascular endothelial cell and isolated mitochondria [28]. Mitochondrial flickers have been linked to calcium movements [17], [24], cytosolic calcium overload [16], [17], and oxidative stress [13], [17], [29]. The present work, in accordance with Cheng's group [10], propose a new model in which flickers are linked to bursts of superoxide in the mitochondrial matrix. However, the two studies disagree on the mechanism. In their pioneer paper, Wang et al. [10] showed that, in cardiac myocytes, inhibiting or silencing the PtP decreases the flash frequency. Their interpretation was that opening of the PtP, which causes mitochondrial depolarization, triggers the flash. Present results show that the mechanism is different in adult skeletal muscle fibers. Superoxide scavenging with tiron decreases the flashes frequency as well as the depolarization frequency, indicating that increase in superoxide level triggers the depolarization and not the opposite. The contradictory results obtained by the two groups could be reconciled by the model proposed in cardiac cells by Zorov et al. [13], [30], according to which, in a single mitochondrion, increased ROS production triggers opening of the PtP, which in turn causes a burst of ROS deriving from the ETC. This phenomenon was called ROS induced ROS release. RIRR is accompanied by a depolarization of the mitochondrion, and in some case preceded by a hyperpolarisation of the same mitochondrion [13]. However, in my work, neither the activation of the PtP by atractyloside nor the inhibition of PtP by cyclosporin A affect the flashes frequency. In rare occasions a “pre increase” in RPmt fluorescence could be identified preceding the flash. Although consistent with the “trigger” ROS production described by Zorov et al [13, fig. 4C], it is far from being a systematic pattern. Still, this could be due to the difference in sampling rate (10 Hz line scan imaging in [13], vs 1 Hz xy imaging here). Indeed, in fig. 4D of [13], which has been realised with a sample rate of 2 Hz in line scan mode, this “Trigger”ROS is barely or not detected. Further work is obviously needed to unravel the mechanism involved in the genesis of flashes.

PtP is a well known redox-sensitive calcium release protein which opening triggers depolarization of mitochondria [31]. Several studies have shown an activation of the PtP by ROS, or more precisely by superoxide [13], [16]. For this reason, despite the fact that PtP is not involved in flash genesis, I investigated the possible involvement of the PtP in the depolarization and calcium release accompanying the superoxide flash. However, inhibiting PtP with cyclosporin A failed to prevent flash-induced depolarization. Cyclosporin A-resistant mitochondrial depolarization induced by ROS has been described in guinea pig cardiomyocytes by Aon et al. [14]. The authors showed that IMAC is involved in this phenomenon. However, inhibition of IMAC failed to affect superoxide production or mitochondrial depolarization in my work. Another possibility would be the uncoupling protein 3 (UCP3), which activation causes a depolarization of mitochondria. Mitochondrial depolarization can trigger a release of calcium from the mitochondrial matrix. Interestingly, UCP3 is activated by superoxide [28]. Obviously, further work will be needed to determine the protein(s) responsible for the depolarization accompanying the spontaneous mitochondrial superoxide flash.

The juxtaposition of active and quiescent units argues against a simple diffusion of superoxide within the cell that would be detected only in mitochondria, where the sensor is. Surprisingly, my results show that, upon metabolic stimulation, increase of superoxide production involves an increase in the number of active units rather than an increase of superoxide production per active unit. In the same way, scavenging superoxide by tiron only slightly decreases flashes amplitude, and most of all dramatically decreases flashes frequency. Neither the number of flashes per unit nor the distribution of intermyofibrillar unit sizes are significantly affected. It is rather the number of active units that decreases. These results raise the question of whether superoxide or one of its derivatives may act as a second messenger between mitochondrial clusters, further stimulating production of superoxide by supplementary units. Such a mechanism, “inter mitochondria” ROS induced ROS release, has already been described in cardiac myocytes [13], [14], [30]. In this model, ROS are released from mitochondria by a channel, either PtP or IMAC, and trigger RIRR in some neighboring mitochondria. Mitochondria behave as a percolation matrix: to be activated, mitochondria have to be near threshold, which could explain the fact that some neighboring mitochondria are devoid of flashes [32]. However, in the present work, altering the activity of PtP or IMAC neither affected the flashes frequency/properties, nor the depolarizations linked to superoxide flashes. Discrepancies with other studies may come from distinct cellular types (skeletal muscle fibers vs cardiac myocytes), or from differences in the recorded events (physiological spontaneous superoxide flashes vs photo-induced toxic propagating ROS increase). Although we can not rule out the hypothesis that mitochondria are activated by a derivative of superoxide which can freely diffuse across membranes (H_2_O_2_ for instance), or that superoxide diffuse to other mitochondria through a different channel, these results suggest that, under physiological conditions, superoxide production is restricted to the productive unit. IMAC or PtP may be involved in pathological propagation of ROS production and mitochondrial activation in skeletal muscle.

To conclude, this work demonstrates a physiological, basal production of superoxide in mitochondria from adult skeletal muscle fibers, that is linked to metabolism. Superoxide production is not homogenous in the fiber, but rather occurs in active mitochondrial units surrounded by quiescent ones. Increase in superoxide production involves activation of quiescent units rather than increase of production per unit. Subcellular localization of superoxide production is of primary importance, as mitochondria are adjacent to calcium release units, containing redox-sensitive calcium signaling proteins [5]–[7], [9]. Numerous evidences support a role of ROS in several muscle functions, including regulation of calcium channels, calcium transporters and calcium-sensing proteins [5]–[7]. Could mitochondrial superoxide locally modulate calcium release unit activity at rest or/and during muscle contraction? Which consequences would have a change in the numbers of active mitochondrial units on calcium signaling? Could it, at some point, affect muscle homeostasis? The present work opens up a novel exciting framework regarding the role of mitochondrial superoxide production in skeletal muscle physiology and pathology.

## Materials and Methods

### Ethical approval

Experiments were performed on 5–8-week-old male OF1 mice (Charles River Laboratories, L'Arbresle, France). All experiments and procedures were in accordance with the guidelines of the French Ministry of Agriculture (87/848) and of the European Community (86/609/EEC). They were approved by the local animal ethic committee of Rhone-Alpes, approval numbers 692660602, 0292.

### Transfection of ratiometric-pericam-mt in adult mice FDB muscles by electroporation

*In vivo* transfection of ratiometric-pericam-mt (RPmt), a kind gift of Dr Miyawaki (RIKEN, Saitama, Japan), was performed within the *flexor digitorum brevis* (FDB) of the animals, according to Di Franco et al. [33]. In brief, 20 µl of 2 mg/ml hyaluronidase dissolved in sterile saline were injected into the footpads of each hind paw of a mouse anaesthetized by isofluorane. 1 hour later, the mouse was anaesthetized by an intraperitoneal injection of a mixture of ketamine and xylazine dissolved in sterile saline (80 and 16 mg kg^−1^, respectively). 15 µl of pcDNA3/RPmt solution (10 µg DNA in sterile saline) were then injected subcutaneously. 10 min later, two electrodes (gold plated stainless steel acupuncture needles) were placed subcutaneously at the starting lines of paw and toes, separated by ∼1 cm. The standard protocol consisted of 20 pulses of 120 V cm^−1^ amplitude and 20 ms duration delivered at a frequency of 1 Hz by a BTX ECM 830 square wave pulse generator (Harvard Apparatus, Holliston, MA, USA). Experimental observations and measurements were carried out 4 to 5 days later.

### Isolation of single FDB fibers

Single fibers were isolated from the mouse *FDB* muscles using a previously described procedure [34]. In brief, mice were killed by cervical dislocation. Muscle were removed and placed in a Tyrode solution containing 0.2% collagenase (Sigma, type 1) for 50 min at 37°C. After this treatment, muscles were kept at 4 C in Tyrode and used within 10 hours. Single fibers were obtained by triturating the muscles within the experimental chamber. For standard observations, fibers were bathed in Tyrode.

### Solution and dye

Tyrode solution contained (in mM): 136 NaCl, 5 KCl, 2.6 CaCl_2_, 1 MgCl_2_, 10 Hepes, 10 Glucose, 5 Pyruvate.

Loading of the dyes were carried out at room temperature in Tyrode. For 2 dyes-detection of mitochondrial superoxide, cells were loaded with 5 µM MitoSox for 10 min. For measurement of mitochondrial membrane potential, cells were loaded with 10 nM Tetramethyl Rhodamine Methyl Ester (TMRM) for 10 min then washed with Tyrode containing 2.5 nM TMRM to avoid loss of the dye. For fluorescence imaging of mitochondrial [Ca^2+^], cells were loaded with 5 µM Rhod 2/AM for 10 min, following a protocol adapted from Hajnoczky et al [35].

TMRM, Rhod-2/AM and MitoSox were from Molecular Probes (Eugene, OR, USA), antimycin A, 4′chlorodiazepam, cyclosporin A, tiron from Sigma (St Louis, MO, USA).

### Confocal imaging and image processing

Experiments were performed at room temperature. Fibers were visualized using a Zeiss LSM 5 Exciter laser scanning confocal microscope (Zeiss, Jena, Germany) equipped with a ×63 oil immersion objective ×1.4 numerical aperture. For detection of RPmt the excitation was provided by an argon laser (477 nm unless otherwise specified) and fluorescence was collected between 505 and 545 nm. For the series of measurements performed with Rhod-2, MitoSox or TMRM, the excitation was from the 543 nm line of a HeNe laser and fluorescence was collected above 560 nm. Fibers were imaged in full frame (xy) mode at a rate of 1 frame/s, corresponding to 1.61 µs per pixel in case of simple staining and 0.8 µs in case of double staining. The zoom factor was set to 2.6, corresponding to a xy pixel size of 0.08 µm. Frame size was 512×512 pixels, with a pixel depth of 12 bit. Pinhole was set to 1 airy unit. For xyz images in fig. 1D, refractive index correction was performed.

For fig. 2B, fibers were visualized using a Leica TCS SP5 AOBS laser scanning confocal microscope equipped with a ×40 oil immersion objective×1.4 numerical aperture, available at the PLATIM of IFR 128 BioSciences Gerland - Lyon Sud. For detection of RPmt, the excitation was provided by a diode laser (405 nm) and an argon laser (491 nm). Fluorescence was collected between 500–550 nm. Rhod-2 was excited with a diode laser (561 nm), and fluorescence collected between 599–683 nm.

### Data processing

Image and data processing were performed using Image/J (NIH, USA) and Microcal Origin (Microcal Software Inc., Northampton, MA, USA).

3D reconstruction of mitochondrial network was performed using Zen software (Zeiss, Jena, Germany).

2D flashes detection was performed on 60–180 frames stacks using a custom-written macro under Image J. In brief, each image of the stack was filtered by a median filter of radius 2 and normalized by a median of the stack. The stack was then temporally smoothed with a factor 4. A new stack of images difference was created by subtracting the image on frame k-1 from the image on frame k. The image of maximal difference was then calculated from the stack of images difference. On this image, the average difference (avg dif) was calculated on areas devoid of flashes. This corresponds to random noise. Flashes were selected by their deviation from the noise, as connected areas that exceeded a threshold defined as avg dif + Cri δdif, where δdif refers to standard deviation, and Cri was 2.5–3.5. Flashing areas smaller than 15 pixels were discarded. A binary mask was created from the selected areas, and applied on the initial stack. Changes in fluorescence in selected areas were expressed as Δ*F/F_0_* or *F/F_0_* where *F_0_* is the resting (or baseline) fluorescence level. An event was considered as a flash if it was at least 10 s long, and its amplitude Δ*F/F_0_* ≥ mean_F_ + 3 δ_F_, where mean_F_ and δ_F_ were the mean value and standard deviation of intensity of fluorescence during the 20 s preceding the flash. Total area of flashing mitochondria was calculated on subsarcolemmal and intermyofibrillar mitochondria, and included mitochondria located on the edge of the picture. Frequency count was performed on intermyofibrillar mitochondria, and excludes areas located on the edges of the picture.

Decay phase of the flash was fitted by a single exponential decay (Microcal Origin, Microcal Software Inc., Northampton, MA, USA). Goodness of fit was verified by a Pearson's chi-square test.

Note: confocal images of time series presented in this paper are projection of the median of the time series stack.

### Statistic

Data were analysed with the software Microcal Origin (Microcal Software Inc., Northampton, MA,USA). Data are reported as mean ± SE. Paired Student's t test, paired Wilcoxon Signed Rank test and Friedman ANOVA were applied, when appropriate. Normal distribution and homogeneity of variance were verified using a Normality test and a F-test for variance. P<0.05 was considered statistically significant.

## Supporting Information

Movie S1 Superoxide flashes in a skeletal muscle fiber. Movie from which fig. 2a is taken. Images were acquired by time-lapse confocal microscopy using a laser-scanning confocal microscope (LSM 5 Exciter; Carl Zeiss, Inc). Frames were taken every second for 3 min and are shown here at 18 Hz. Images were filtered by a median filter of radius 2.
(4.25 MB AVI)
